# Soziologie in Neuseeland und in Argentinien

**DOI:** 10.1007/s11577-021-00796-2

**Published:** 2021-09-27

**Authors:** Heike Delitz

**Affiliations:** Fachgruppe Soziologie, Otto-Friedrich-Universität Bamberg, Feldkirchenstraße 21, 96052 Bamberg, Deutschland

## *Blois*, *Juan Pedro*:

Sociology in Argentina. A Long-Term Account. Cham: Palgrave Macmillan 2020. 154 Seiten. ISBN: 978-3-030-63519‑0. Preis: € 53,49.

## *Crothers, Charles*:

Sociologies of New Zealand. Cham: Palgrave Macmillan 2018. 151 Seiten. ISBN: 978-3-319-73866‑6. Preis: € 53,49.

Innerhalb der Reihe „Sociology Transformed“ stehen diese beiden Bände für differente gesellschaftliche Kontexte, für andere Schreibstile und Selbstverständnisse von Soziologie. Charles Crothers erzählt die „Geschichte“ der neuseeländischen Soziologie in Gestalt von Auflistungen – von Institutionen, Themen und Subdisziplinen, Personen, Studierendenzahlen, Journals. Juan Pedro Blois dagegen setzt die (weit dramatischere) Geschichte der argentinischen Soziologie und ihrer Institutionen zutiefst in deren Kontext: in den Kontext politischer Ereignisse und Gewalt, in den Kontext staatlicher, militärischer, studentischer und wissenschaftlicher Politiken.

## Neuseeland: Abhängigkeit von und Soziologie jenseits des „Mainstreams“

Die Darstellung der Soziologie Neuseelands verzichtet weitgehend auf historische Kontexte; sie präsentiert diese Soziologie als eine, die durch Spezialisierungen, neue Themen, Ressourcen, Personen und Institutionen gekennzeichnet ist. Diese „Geschichte“ darzustellen sei deshalb interessant, weil die neuseeländische Gesellschaft an der globalen Peripherie und deren Soziologie im Konflikt mit dem soziologischen „Mainstream“ steht. Eingangs wird versprochen, dass auch „Schlüsselmerkmale der neuseeländischen Gesellschaft“ zur Sprache kommen; auch wird eine Darstellung in vier „Phasen“ (viii) angekündigt. Tatsächlich folgt die Einteilung einer Unterscheidung zwischen der „vordisziplinären“ Phase (bis 1960, Kap. 2), der „empirizistischen“ (Kap. 3), der „spezialistischen“ (Kap. 4) und der „zeitgenössischen“ Phase (Kap. 5). Diese letzten drei Phasen sind allerdings nicht datiert. Insgesamt wird allenfalls minimal chronologisch erzählt: In Kapitel 3 geht es um alle Departments, in Kapitel 4 um alle Spezialisierungen und im fünften Kapitel um Fördereinrichtungen, Netzwerke usw. Insgesamt entsteht ein Bild, nach dem das neuseeländische sozialwissenschaftliche Wissen zuerst „diffus lokalisiert“ gewesen sei, sich dann disziplinär eingeteilt habe, dann spezialisierte und heute in „semi-silos“ wiederum disziplinär geschlossen sei (viii).

Zur Geschichte Neuseelands insgesamt erfährt man nur so viel: Neuseeland gehöre zu den Siedlungskolonien. Die koloniale Gewalt, die Eindringung englischer und europäischer Immigranten (Pākehā) bleibt unsichtbar. Es folgen nur Stichpunkte: „Maori settlement; Early exploration and then settlement by whalers, sealers, missionaries, and traders; Official setting-up of a Pākehā state …“ (S. 10). Die restliche Darstellung der „Geschichte“ ist ganz auf die Soziologie reduziert (Kap. 2): Das vordisziplinäre Wissen sei zerstreut und auf Maori und Polynesier konzentriert gewesen; dagegen beginne die Soziologie 1921 mit der Institutionalisierung zunächst als Hilfsfach, dann als zunehmend organisierte Erforschung von Gemeinden, Bevölkerungsproblemen, Wohnungsbau und Familien. Auch wird erwähnt, dass Karl Popper von 1937 bis 1946 in Neuseeland lehrte (S. 31). In Kapitel 3 wird die Geschichte aller sieben soziologischen Departments skizziert – obgleich der Autor darauf den Schwerpunkt legt, genügt für die Besprechung diese Information. Es werden je Jahreszahlen und Personen genannt, erste Abschlüsse und Veränderungen der Curricula etc. Es wird für die Soziologieabsolventinnen und -absolventen von einer „feminisation and Pākehā-isation“ (S. 40) der Disziplin gesprochen. Kapitel 4 listet Forschungsgebiete und Nachbardisziplinen auf. Erwähnt werden Demografie, Familiensoziologie, die Erforschung ethnischer Minderheiten, „area und community studies“, Soziologie sozialer Ungleichheit, Bildung und Wahlverhalten, Gender, Polynesian/Māori und „cultural studies“, Soziologie von Freizeit und Sport, Politik und Wirtschaft, Gesundheit und Medizin, Agrar‑, Stadt‑, Religions- und Umweltsoziologie, Soziologie von Sexualität, Schlaf, Sterben, Tod. Als neuere Trends werden „area und ethnic studies“ hervorgehoben – und die Verlagerung von der Pākehā-Kultur zu Māori, Pacific, Asian, African Studies. Zudem werden Rassismus und Rugby, Migration, feministische und gesundheitssoziologische Forschungen als Trends genannt. Genauer skizziert werden u. a. Māori und Pacific Studien, sowie auch Nachbardisziplinen. Was die Māori Studies betrifft (S. 76ff.), so seien zunehmend Māori die Autorinnen und Autoren; erwähnt wird auch, dass soziologische Konferenzen mit Māori-Ritualen beginnen. Das Potenzial der neuseeländischen Soziologie liege hier in der Ernstnahme des indigenen Wissens, die indes erst zu leisten sei. Dagegen (Kap. 5) ist der Einfluss der internationalen Soziologie groß, ebenso wie der *brain drain* der neuseeländischen Soziologie. Es werden prominente Gäste erwähnt (Parsons, Wacquant, Burawoy), einflussreiche Theorien (Funktionalismus, Weber, Marxismus, Poststrukturalismus) – in einer von Theorien nur „lightly touched“ Soziologie (S. 115). Es werden Einführungsbücher und Methoden genannt (u. a. ein Ansatz, der „indigene“ Forschungsmethoden benutzt).

Das Fazit lautet: Neuseeland könnte angesichts seiner Spezifik (Siedlungskolonie, „small-scale-society“, Insellage, neoliberales Experiment) eine eigene Soziologie hervorbringen (S. 131). Es fehle aber das Interesse an den indigenen Gesellschaften, sowohl der des Pazifiks als auch der Hauptinsel oder interethnischer Kollektive. Stattdessen ende die Soziologie Neuseelands in „agribusiness, gender, sports, and health sociology“ (S. 132).

## *Argentinien*: *„A Southern Sisyphus“*

Das ist im Fall Argentiniens ganz anders. Die Darstellung dieser Soziologie folgt den enormen politischen Kontrasten, denen diese Gesellschaft im 20. Jahrhundert unterlag: Zwischen 1930 und 1976 gab es sechs Militärputsche; Demokratien wechselten mit Diktaturen. Seit 1983 eine Demokratie, unterliegt Argentinien weiter Krisen. Phasen von Hyperinflation und Arbeitslosigkeit wechseln sich mit solchen der Stabilität ab – die argentinische Gesellschaft insgesamt erscheint damit als „enigma“: „it was possible for Argentinia to be the most advanced welfare state in Latin America … in the 1950s, the cruelest dictatorship in the 1970s … and take a neoliberal turn in 1990s“ (S. 2). Die Geschichte der Soziologie war ebenso „troubled“. Sie wird in sieben Kapiteln erzählt. Nach der Einführung geht es im zweiten Kapitel um die Instituierung einer „modernen“ Sozialwissenschaft durch Gino Germani (1955–1966), um die Politisierung und die Entstehung einer – gegen diese gerichtete – „Nationalen Soziologie“ (1966–1974) im dritten Kapitel. Weiter geht es um die Soziologie unter der Militärdiktatur (1974–1983; Kap. 4), um die Rückkehr von Demokratie und Soziologie (1983–1989; Kap. 5) sowie um die Professionalisierung seit 1989 im sechsten Kapitel, gefolgt von einem kurzen Fazit. Auch in diesem Buch geht es um Studierendenzahlen, Departements und Personen; und doch wird diese außereuropäische Soziologie viel plastischer in dem, was sie antreibt, wo die Konflikte liegen, wie tief Gesellschaft, Politik und Wissenschaft verknüpft sind.

Einführend wird diese Soziologie in ihrer Bedeutung für Lateinamerika betont: Viele Begriffe (Populismus, Marginalisierung, Zentrum, Peripherie) entstammen Argentinien. Zugleich wird diese Soziologie durch die „zigzagging history“ enorm beeinflusst (S. 4). Ähnlich wie der Arbeit zur neuseeländischen Soziologie geht es um eine Soziologie, die unter internationalem Einfluss steht, aber um eine, die sich stärker im Konflikt zwischen internationaler und nationaler Orientierung entfaltet. Auch hier geht es um die Inhalte der Soziologie als Disziplin, zentriert aber um Kämpfe um diese und bezogen auf die politische Situation. Unter den Instituten wird vor allem das der Universität Buenos Aires (UBA) hervorgehoben: Die Soziologie wird als eine erzählt, die im Jahr 1959 an der UBA beginnt (Kap. 2). Gefördert durch ausländische Geldgeber und orientiert an der US-Soziologie ging es Germani darum, die seit 1898 bestehende *sociólogos de cátedra *aufzulösen. Auf eine „wissenschaftliche“ Soziologie zielend, lehnte Germani jegliche Kooperation ab (S. 23). Er war daher gezwungen, Nicht-Soziologen einzustellen. In der Durchsetzung der positivistischen Soziologie kamen ihm politische Ereignisse zugute: die Absetzung Peròns 1955, aber auch dessen vorherige Ersetzung liberaler durch konservative Professoren erlaubten Germani rigide Eingriffe in die Universität. Der „Kollaboration mit dem Tyrannen“ bezichtigt (S. 24) wurden die, die im Peronismus lehrten, durch jene ersetzt, die im Untergrund unterrichteten. Germani konnte seine Vorstellungen von Soziologie in die Curricula festschreiben. Bedeutend wurde er dank seiner Arbeit zum Peronismus, dessen Synthese linker und rechter Motive ein Rätsel schien oder schlicht als Faschismus. Germani dagegen machte die Spezifik des Peronismus (Zensur plus „Demokratie“, d. h. Plebiszite) und dessen Träger deutlich: die neue Arbeiterschicht aus der Provinz, die als solche empfänglich war für eine autoritäre Politik. Fast nur englische Literatur lehrend, erzeugte Germani indes zunehmend ebenso den Unmut der Studierenden, wie deren Politisierung durch die Revolution in Kuba, die sie dazu führte, jede objektivistische Soziologie abzuwehren. Im Zeitraum von 1966 bis 1974 (Kap. 3) schlug die Soziologie eine gegenüber sowohl Germani als auch den Studierenden entgegengesetzte Richtung ein: Die Militärregierung bekämpfte die Studierenden ebenso wie eine importierte Soziologie. *Catedras nacionales *wurden mit der Aufgabe eingerichtet, für die „nationale und soziale Befreiung“ Partei zu ergreifen. Mao, Ho Chi Minh, Bolivar, Peròn wurden zu autoritären Texten und von katholischen und konservativen Autoren gelehrt. Trotzdem schritt die Politisierung der Studierenden voran (S. 47). Weder Positivismus noch Funktionalismus oder Marxismus waren aus ihrer Sicht „adäquat“. In der Dritten Welt, in der der Hauptkonflikt die imperiale Herrschaft der USA gegenüber den ausgebeuteten Nationen war, schien ihnen nur die „peronistische“ Soziologie angemessen. In der Rückkehr Peròns 1973 verkündete die Regierung eine „Kulturrevolution“. Die Politisierung intensivierte sich weiter. Kritiken an den – von Germani ins Zentrum gestellten – Politiken der Modernisierung und „Entwicklung“ wurden entfaltet. Nach Peròns Tod (1974, Kap. 4) beginnt unter dem Druck rechtsextremer Parteien, die aus der Krise (der Ökonomie und der Konflikte zwischen rechtem und linkem Flügel) Gewinn schlagen, die „Säuberung“ der Universitäten. Soziologie-Institute werden geschlossen, Soziologinnen und Soziologen gehen ins Exil. Zunächst überziehen Paramilitärs das Land mit Terror, dann – umso brutaler – die Junta. Antiimperialistische Bücher werden als „links“ zensiert. Zahlreiche Soziologinnen und Soziologen gehören zu den *Desaparecidos* (S. 72). In Seminaren arbeiten verdeckte Offiziere, es werden thomistische Philosophen und „racist German authors“ gelesen (S. 72, Fn.). Bis 1981 wird jede Forschung untersagt. Gleichwohl beenden 2200 Studierende der UBA im Jahr 1978 ihr Studium – in einen inexistenten Arbeitsmarkt hinein. Neben dem Verlust jeglichen Vertrauens in den Staat leidet Argentinien an Inflation und Deindustrialisierung. Als nach der missglückten Eroberung der Falkland-Inseln das Regime den Weg für Wahlen freimacht, werden die Herausforderungen enorm. Die neue Demokratie (1983–1989, Kap. 5) verändert die Soziologie erneut. Die ökonomische Krise erschwert deren Institutionalisierung, die Studierenden rebellieren weiter gegen die positivistische und auch gegen angewandte Soziologie. Erneut gibt es Attacken gegen „Kollaborateure“. Englischsprachige Literatur wird verfemt. Die politischen Ziele der Studierenden – eine Soziologie „für die Unterdrückten“ – bleiben aber unerreicht. Die Exilanten lehren nun eine Soziologie, die auf die Stärkung demokratischer Haltung zielt. Gelesen werden Crozier, Habermas, Luhmann, Touraine (S. 104ff.) und nur wenige lateinamerikanische Soziologien (S. 109). Es gibt gleichwohl weiter *cátedras nacionales *und insofern nach wie vor die Konfrontation zwischen „wissenschaftlicher“ und „essayistischer“ Soziologie. Seit 1989 (Kap. 6) bleibt die wirtschaftliche und politische Lage instabil. Weder in den 1990er-Jahren (unter neoliberalen Bestrebungen) noch danach scheint die Disziplin politisch wichtig. Sie nimmt nun einen „practical turn“ (S. 118), wird angewandte Wissenschaft.

Im Fazit wird die untrennbare Beziehung zwischen dem Aktivismus der Studierenden, der Politik und der (De‑)Institutionalisierung der Soziologie betont sowie die Situation einer Soziologie an der „Peripherie“, die sich für die Theoriezentren (Europa, USA) öffnet *und* eine „nationale“ oder lateinamerikanische Theorie sucht. Mit Fernandez – einem Juristen und Dozenten der UBA – ist seit 2019 erneut ein peronistischer Politiker Präsident, der indes die Wissenschaften fördert. Dem steht seit Covid-19 eine neue Wirtschaftskrise entgegen, gefolgt von einer neuen Polarisierung, in der die Soziologie nun auf der Seite des Staates steht – und erneut angegriffen wird. In dieser Situation ist es gerade die dargestellte „Zickzack“-Geschichte, die dem Autor Hoffnung macht.

Beide Bände sind interessant aus mindestens drei Gründen: zunächst im Blick auf die Situation von außereuropäischen Soziologien, im Blick auf die Spannung zwischen lokalen Autoren und Zielen und der internationalen soziologischen Theorie und Methodologie. Es wird zweitens im Band zu Argentinien deutlich, wie stark die Soziologie politisch ist (auch als positivistische) und wie sehr politisch abhängig. Die Wechselhaftigkeit der Geschichte, die Brutalität, mit der diese Soziologie konfrontiert war, ist unbedingt erzählenswert. Drittens wird – gerade im Band zu Argentinien – auch deutlich, wie sehr die Disziplin auch in diesem Fall, auch in der Suche nach einer „nationalen“ (oder lateinamerikanischen) Soziologie, eine westliche Disziplin bleibt. Mit anderen Worten, was auffällt ist, wie wenig auch die außereuropäischen Soziologien von post- oder/und dekolonialen Perspektiven berührt sind. Das ist gerade im Blick auf Argentinien bemerkenswert, denn sowohl post- als auch dekoloniale Kritiken kommen nicht zuletzt aus Lateinamerika (*Latin American Subaltern Studies Group*, A. Quijano), auch aus Argentinien (W. Mignolo) – und gerade Mignolo konzentriert sich dabei auf die Kritik der Delegitimierung des indigenen Wissens, auf die epistemische Gewalt. Dass diese Gewalt auch mit der Soziologie – zumal mit einer positivistischen – einhergeht, wird weder im Band zu Argentinien noch zu Neuseeland adressiert. Zugleich ist hier das Problem zumindest erkannt. Wenn letztlich nur nebenbei, so betont Crothers doch als Potenzial der neuseeländischen Soziologie gerade die Anerkennung des indigenen Wissens und der indigenen Modi kollektiver Existenz. Wie erwähnt, ist diese Lücke – die fehlende Selbstkritik – gerade im Band zu Argentinien auffällig, wo zwar der Kampf zwischen einer national und international orientierten Soziologie betont wird, aber nicht nur indigene Stimmen und Kollektive vollkommen unsichtbar bleiben, sondern auch die postkoloniale Theorie vollständig fehlt (ebenso wie nahezu jede Nachbardisziplin). Trotz dieser bezeichnenden Lücke im Blick auf Kolonisierung und Kolonialität des Wissens – in den außereuropäischen Soziologien selbst – und trotz der unbefriedigenden Weise, in der die neuseeländische Soziologie erzählt wird (im fast völligen Fehlen von Geschichte, der Auflistung von Institutionen, Personen, Texten, Themen) sind beide Bände informativ – in einer insgesamt wichtigen Reihe über Soziologie weltweit.

